# Nutrigenomic regulation of one-carbon metabolism and the circadian clock in health and disease

**DOI:** 10.1093/jb/mvag050

**Published:** 2026-07-01

**Authors:** Jean-Michel Fustin

**Affiliations:** The University of Manchester; Faculty of Biology, Medicine and Health; Centre for Biological Timing; The Meth Lab; Oxford Road, Manchester, M13 9PL, UK

**Keywords:** circadian clock, epigenetics, epitranscriptomics, one-carbon metabolism, S-adenosylmethionine

## Abstract

Methylation of DNA, histones and RNA is central to the regulation of circadian rhythms, yet the biochemical origin of the methyl groups driving these modifications has received comparatively little attention in circadian biology. This review explores the bidirectional crosstalk between the methyl cycle and the mammalian circadian clock. We describe how S-adenosylmethionine-dependent epigenetic and epitranscriptomic modifications constitute essential layers of circadian gene regulation, and how the clock orchestrates the rhythmic expression of one-carbon metabolism enzymes and oscillations in S-adenosylmethionine availability. The direct interaction between the *S-adenosylhomocysteine hydrolase* and the core clock component BMAL1 at circadian gene promoters emerges as a molecular nexus linking methyl group supply to clock-driven transcription. We further discuss how the methyl cycle occupies a privileged position within the circadian entrainment hierarchy, acting as both a target of nutritional zeitgebers in peripheral tissues and a potential source of metabolic feedback to the central pacemaker, and how dietary perturbation of the methyl cycle disrupts circadian rhythms. Finally, we discuss how this crosstalk is implicated in metabolic liver disease, cancer, neurological disorders and aging. Together, these findings position the circadian clock as a sensitive read-out of nutritional methyl metabolic status, with broad implications for chronobiology and nutrigenomics.

## Graphical Abstract

**Graphical Abstract:**
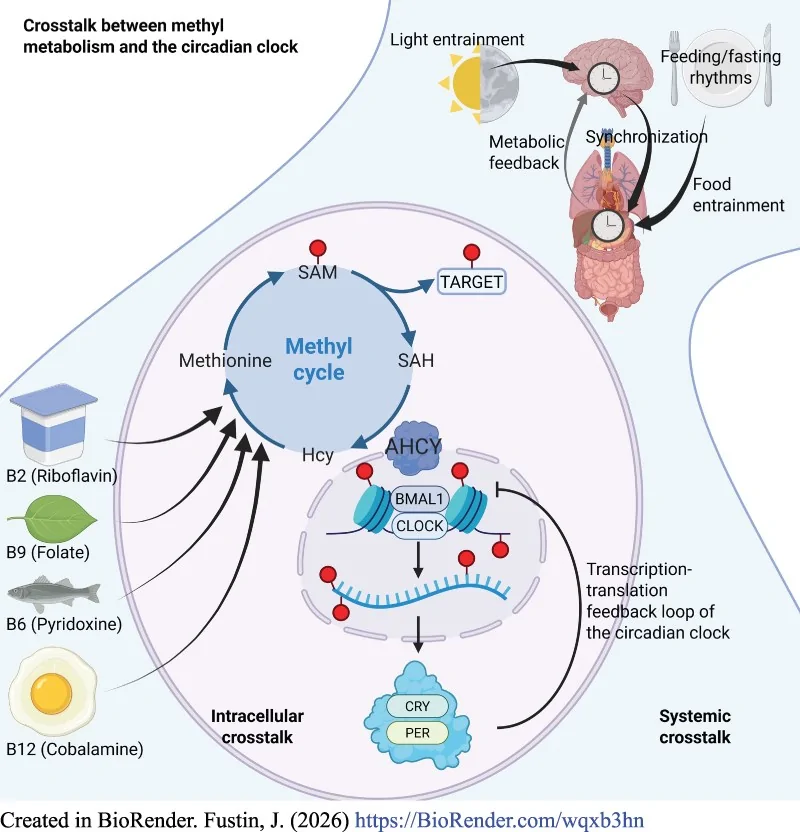

## The Circadian Clock—A Multilayered Regulatory System

### The core transcriptional–translational feedback loop

Circadian rhythms are self-sustaining, ~24-hour oscillations that govern virtually every aspect of mammalian physiology, from sleep–wake cycles and hormone secretion to immune function and metabolism. At the molecular level, they rely on clock genes in an autoregulatory transcriptional–translational feedback loop (TTFL) that are remarkably conserved across eukaryotes. In mammals, the positive arm of this loop is driven by the bHLH–PAS transcription factors CLOCK and BMAL1, which heterodimerize, translocate to the nucleus and bind E-box sequences (CACGTG) in the promoters of other clock-genes as well as clock-controlled genes (CCGs) to activate their transcription; this occurs predominantly during the day in the liver (*1*). Among the most important clock genes are the Period genes (Per1–3) and Cryptochrome genes (Cry1–2), whose protein products form PER–CRY repressor complexes that translocate back to the nucleus and inhibit CLOCK-BMAL1, thereby suppressing their own synthesis (*1*). As repressor proteins are degraded, CLOCK-BMAL1 activity is restored and a new cycle begins. Secondary interlocking loops composed of other clock genes not only provide further stabilizing oscillations of the main oscillator but also drive the expression of CCGs at different phases during the day, e.g. the loop formed by the nuclear receptors REV-ERBα and RORα, which are also CLOCK-BMAL1 targets, acting on ROR Response Elements (ROREs) in the promoter of target genes (including *Bmal1* itself) that REV-ERBα represses and RORα activates, the latter occurring during the night in the liver (*1*).

A major determinant of the period of the TTFL is the timing of clock protein degradation. Casein kinase 1δ/ε (CK1δ/ε) phosphorylates PER proteins, targeting them for ubiquitination and proteasomal degradation; AMPK phosphorylates CRY proteins via an analogous mechanism (*1*). These post-translational events introduce the delay necessary to convert rapid transcriptional oscillations into a 24-hour period and represent key points at which metabolic inputs (including the methyl cycle, as discussed in ‘Crosstalk between One-carbon Metabolism and the Circadian Clock’) can modulate clock speed. Beyond phosphorylation, and like most transcriptional programmes, the clock is further shaped by ubiquitination and sumoylation of clock proteins (*1*). These post-translational modifications (PTMs) determine clock protein stability and nuclear localization and are covered in detail elsewhere (*2*); their relevance here is to underscore that the clock integrates metabolic signals simultaneously at multiple regulatory tiers, and that perturbation of the methyl cycle can impinge on timing through both epigenetic and post-translational mechanisms.

In mammals, a master pacemaker in the suprachiasmatic nuclei (SCN) of the hypothalamus is entrained by the light–dark cycle and synchronizes peripheral clocks in virtually every organ. Peripheral clocks are additionally responsive to feeding, temperature and hormonal signals, making them directly sensitive to nutritional inputs that are independent of the light cycle (*1*).

### Epigenetic regulation: histone modifications

The TTFL alone cannot account for the tissue-specific, environmentally responsive and rhythmically precise expression of clock genes and CCGs. This requires the coordinated remodelling of chromatin over a 24-hour cycle, named the circadian epigenome (*3*).

Rhythmic histone acetylation was among the first epigenetic marks linked to circadian transcription (*4*). CLOCK itself possesses intrinsic acetyltransferase activity targeting H3K9/14 and also acetylates BMAL1, a modification required for CRY1 recruitment to the repressor complex (*5**,*  *6*). The NAD^+^-dependent deacetylase SIRT1 opposes CLOCK-mediated acetylation by deacetylating BMAL1, PER2 and H3K9/14 at circadian promoters, linking clock amplitude directly to cellular energy status via the NAD^+^/NAMPT feedback axis (*7**,*  *8*).

Histone methylation is equally central to circadian chromatin dynamics and is a major interface between the methyl cycle and the clock. H3K4 trimethylation (H3K4me3), a hallmark of active promoters, oscillates in a circadian manner through the action of a recruited histone H3K4 methyltransferase to CLOCK–BMAL1 complexes and generates a chromatin state permissive for transcriptional activation (*9*). Loss of MLL1 abolishes both rhythmic H3K4me3 and downstream H3K9/14ac, establishing histone methylation as hierarchically upstream of acetylation in the circadian chromatin programme (*9*). The repressive phase is mediated by EZH2-deposited H3K27me3 and by H3K9 methylation during transcriptional silencing (*10**,*  *11*). Lysine demethylases balance this cycle, removing activating and repressive methyl marks in a phase-dependent manner (*12**,*  *13*).

### DNA methylation

Under basal conditions, DNA methylation at CCG promoters is relatively stable, though genome-wide cytosine modifications, including 5-hydroxymethylcytosine, display circadian oscillations across other genomic regions (*14**,*  *15*). At the pathological level, promoter hypermethylation of PERs and BMAL1 has been documented across multiple cancers, establishing a link between epigenetic clock silencing and oncogenesis (*16*).

### Epitranscriptomic regulation: RNA methylation

Biochemical modification of mRNA (collectively termed ‘the epitranscriptome’) represents a more recently appreciated regulatory layer whose dynamic nature is still being actively debated. Unlike histone methylation, whose writers, erasers and readers are relatively well characterized, the field of RNA methylation has expanded rapidly only since high-throughput mapping of mRNA modifications became possible in the early 2010s. In mammals, at least two distinct S-adenosylmethionine (SAM)-dependent mRNA methylation systems are relevant to circadian regulation: internal N^6^-methyladenosine (m^6^A) and the 5′ mRNA cap.

The most abundant internal modification is m^6^A, catalysed by the core methyltransferase complex METTL3/METTL14 and the adaptor WTAP. A related N^6^-adenosine-methyltransferase, METTL16, provides a direct mechanistic link between SAM availability and gene expression: when SAM is abundant, METTL16 methylates the *Mat2a* pre-mRNA, leading to intron retention and premature decay; when SAM is limiting, the absence of methylation permits normal splicing, increasing MAT2A protein and SAM production. This constitutes an elegant negative feedback loop in which a SAM-dependent RNA methyltransferase directly senses and restores cellular methyl group supply (*17–20*).

m^6^A influences mRNA splicing, stability, nuclear export and translation, decoded by reader proteins of the YTH domain family that promote distinct, and sometimes opposing, functional outcomes (*21*). Whether m^6^A is truly dynamic, deposited and removed in response to physiological signals like histone methylation, or whether apparent changes reflect mRNA turnover rather than active mark cycling, remains actively debated; two demethylases, FTO and ALKBH5, have been characterized but their physiological targets and timescales of action are not yet fully resolved (*22**,*  *23*).

A distinct but equally SAM-dependent methylation occurs at the 5′ cap. The RNA guanine-N^7^ methyltransferase RNMT deposits a 7-methylguanosine (m7G) cap essential for mRNA stability, splicing and ribosome recruitment, and whose degree of methylation modulates the translational efficiency of individual transcripts (*24**,*  *25*). Adjacent 2′-O-ribose methylations (cap-1 and cap-2), catalysed by CMTR1 and CMTR2, further influence splicing, processing and translation (*26*). No eraser has been described for either m7G or 2′-O-ribose in mammals. While cap methylation was long considered a constitutive modification, it is now clear that it can be induced and is dynamic across biological timescales (*24**,*  *25*).

**Fig. 1:**
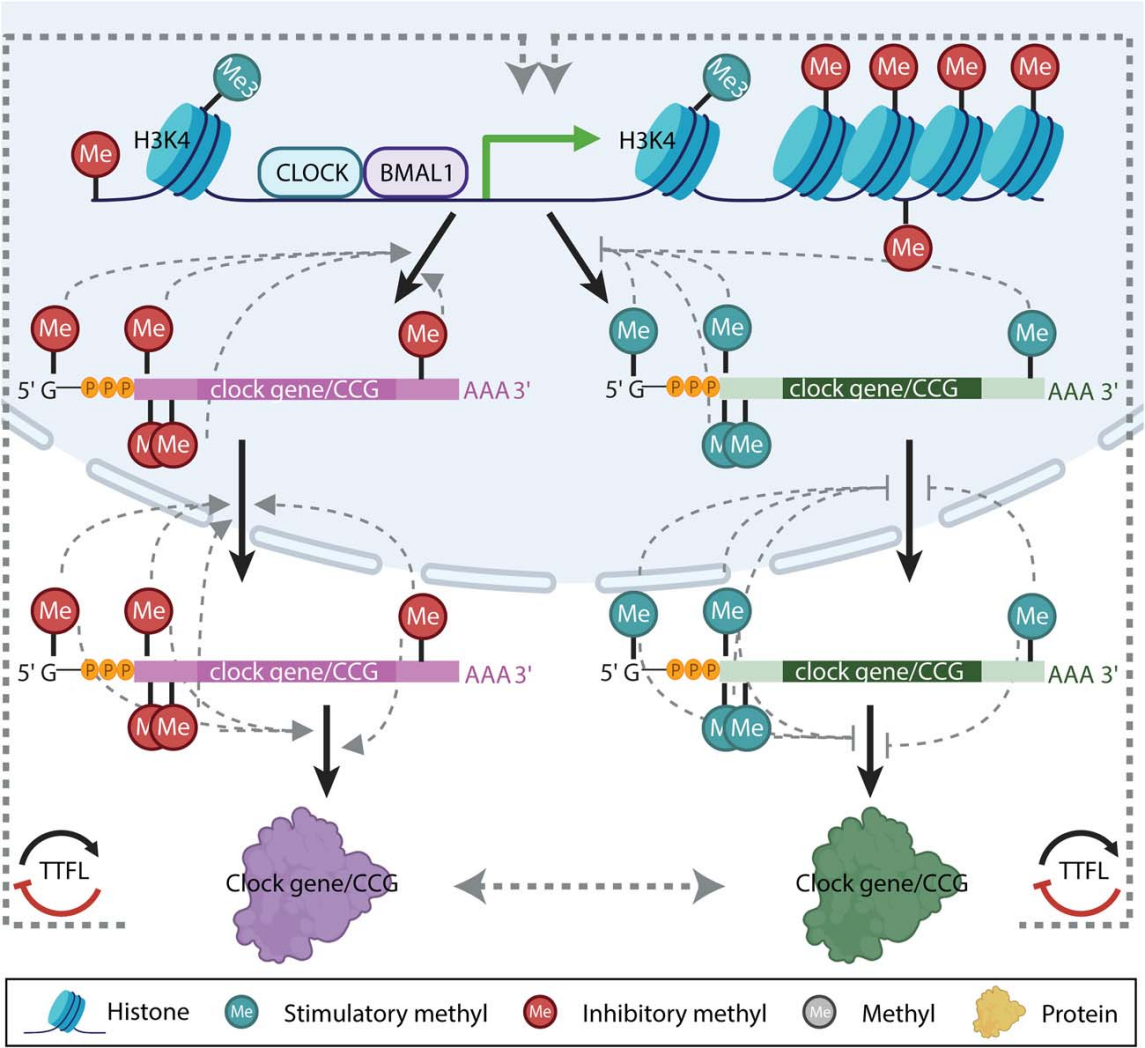
**Pervasiveness of methyl groups within the TTFL.** The TTFL of the clock is driven by CLOCK-BMAL1 stimulating the expression of other clock genes, directly or indirectly via accessory loops, that in turn repress or activate the transcription of target genes, including CLOCK-BMAL1 targets themselves. This TTFL is itself submitted to the influence of various DNA, mRNA (cap or internal) and histone methyl marks that can either have an inhibitory or stimulatory effect on the expression of clock and CCGs. Methylation of rRNA and tRNA and non-histone proteins, not shown here, likely also have a role to play. Figure elements from BioRender. Fustin, J. (2026)

Depending on the transcript, local sequence and structure, and cell type, any mRNA methyl mark may have stimulatory or inhibitory effects on transcription, splicing, nuclear export, translation and stability. Fig. 1 illustrates how epigenetic and epitranscriptomic methylation are an integral part of the circadian TTFL.

Beyond mRNA, SAM-dependent methylation is also pervasive in ribosomal RNA (rRNA) and transfer RNA (tRNA), where it plays essential roles in translation fidelity and efficiency. In rRNA, over 100 methylation events stabilize ribosome structure and modulate translational accuracy and output (*26*). In tRNA, methylations at multiple positions, including m^5^C and m^1^A by, are essential for tRNA folding, stability and decoding (*26*). Given that ribosome biogenesis and translational capacity fluctuate across the circadian cycle, and that clock proteins must be synthesized with precise timing, SAM-dependent rRNA and tRNA methylation represents potentially important but largely unexplored the link between methyl group supply and circadian protein output.

## One-Carbon Metabolism—The Biochemical Engine of Methyl Group Supply

### SAM: the universal methyl donor

SAM is the principal biological methyl donor, participating in more than 200 distinct methyltransferase reactions in the human cell (*27*), and is widely recognized as one of the most widely used enzyme cofactors in cellular metabolism (*28*). SAM is synthesized in the cytoplasm from the essential amino acid methionine and ATP by methionine adenosyltransferase (MAT), in a reaction that consumes all three phosphate groups of ATP and is essentially irreversible under physiological conditions (*28*). The liver is the principal site of SAM metabolism, accounting for approximately half of daily methionine processing and ~85% of all transmethylation reactions (*28*).

Three genes encode the catalytic subunits of MAT in mammals: MAT1A, a catalytic subunit expressed in differentiated hepatocytes; MAT2A, the catalytic subunit expressed in all extrahepatic tissues and encoding a lower-affinity isoform; and MAT2B, a regulatory subunit for MAT2A (*28*). This isoform pattern is developmentally regulated: foetal liver expresses MAT2A, which gives way to MAT1A after birth (*28*) and is pathologically reversed in liver injury and hepatocellular carcinoma (*29**,*  *30*), with direct consequences for SAM levels and epigenetic stability discussed in ‘Implications for Health and Disease’.

### The transmethylation–SAH–homocysteine axis

After donating its methyl group to a substrate, SAM is converted to S-adenosylhomocysteine (SAH). This by-product is a potent competitive inhibitor of virtually all SAM-dependent methyltransferases, binding their active site with similar affinity to SAM but without transferring a methyl group (*28*). The ratio of SAM to SAH is therefore the primary biochemical determinant of methyltransferase activity in vivo. SAH is hydrolysed to homocysteine and adenosine by SAH hydrolase (AHCY); this reaction thermodynamically favours SAH synthesis and proceeds in the hydrolysis direction only because both products are rapidly removed (*28*). AHCY is not merely a housekeeping enzyme. It directly interacts with partners at sites of dynamic histone methylation, including the polycomb repressive complex 2 (*31**,*  *32*), and BMAL1 at circadian gene promoters (*33*), making it a molecular nexus between methyl metabolism and the clock. Whether AHCY also colocalizes with other methyltransferase remains to be confirmed, but AHCY has been shown to interact with FTO and protect mRNA from m6A demethylation (*34*), and large interactome studies suggest AHCY interacts with the translation initiation complex (*35*), which is largely dependent on cap methylation.

Homocysteine sits at a metabolic junction. It can be remethylated back to methionine via two routes: methionine synthase (MTR), which requires 5-methylTHF and vitamin B12, operating in all tissues; or betaine–homocysteine methyltransferase (BHMT), which uses betaine derived from choline and is restricted to liver and kidney (*28*). Alternatively, in the liver, brain and kidney, homocysteine can enter the transsulfuration pathway—converted to cysteine via cystathionine β-synthase (CBS) and cystathionine γ-lyase, both requiring vitamin B6—ultimately contributing to glutathione synthesis and cellular antioxidant capacity. SAM itself allosterically governs this partitioning: at high SAM, MTHFR is inhibited (reducing 5-methylTHF for remethylation) and CBS is activated (promoting transsulfuration); at low SAM, the brake on remethylation is released and homocysteine is efficiently recycled to restore the methionine pool (*28*).

### The folate cycle and dietary methyl group sources

The methyl group of methionine originates primarily from the folate cycle. Natural dietary folate (vitamin B9) is mainly 5-methyltetrahydrofolate (5-MTHF), the biologically active form of folates that can be absorbed into the bloodstream. 5-MTHF is however highly unstable, and many countries have mandated the fortification of flour and cereals with the synthetic folic acid (*36*). Folic acid must be reduced to tetrahydrofolate (THF) and acquires one-carbon units principally from serine via serine hydroxymethyltransferase (SHMT, requiring vitamin B6), generating 5,10-methyleneTHF. MTHFR (requiring riboflavin, vitamin B2) irreversibly reduces this to 5-methylTHF, whose methyl group is then transferred to homocysteine by MTR (requiring vitamin B12) to regenerate methionine (*28**,*  *37*).

Choline is a particularly important alternative methyl source: oxidized to betaine, it supports BHMT-mediated remethylation in the liver and kidney independently of folate. The key nutritional implication is that adequate SAM levels require a coordinated supply of at least six essential dietary components: methionine, folate (B9), cobalamin (B12), riboflavin (B2), pyridoxine (B6) and choline/betaine. Deficiency in any one impairs the cycle at a specific point and reduces methylation capacity, with downstream consequences for all SAM-dependent reactions including those at circadian gene promoters (*38*).

### Homeostatic regulation and SAM as a metabolic signal

The liver maintains SAM within a tight physiological range through the glycine N-methyltransferase (GNMT) buffer. GNMT, present at 1–3% of hepatocyte cytosolic protein, transfers excess SAM methyl groups to glycine to produce sarcosine, and is itself inhibited by 5-methylTHF—creating a direct reciprocal link between the folate cycle and SAM homeostasis (*28*). Genetic disruption of this buffer is instructive: GNMT-knockout mice accumulate ~40-fold excess hepatic SAM and develop global hypermethylation, NASH and hepatocellular carcinoma; Mat1a-knockout mice suffer chronic SAM deficiency and develop the same pathological endpoint through hypomethylation—demonstrating that both deficiency and excess of SAM are harmful (*28*).

A central insight for this review is that SAM and the SAM/SAH ratio function not merely as passive substrates but as active metabolic signals that communicate nutritional state to the epigenome. Because many methyltransferases have kinetic parameters in the range of physiological SAM and SAH concentrations, small dietary-driven fluctuations in the SAM/SAH ratio are sufficient to alter specific histone and DNA methylation patterns without any change in methyltransferase expression (*39**,*  *40*). Methionine restriction produces rapid, reversible decreases in H3K4me3 at specific loci within hours; restoration of methionine reverses these marks (*39*). Critically, intracellular methionine and SAM concentrations themselves oscillate in a circadian manner in the mouse liver (*41*), providing the metabolic foundation for the bidirectional clock–methyl metabolism crosstalk explored in ‘Crosstalk between One-carbon Metabolism and the Circadian Clock’.

A further layer of metabolic interconnection exists between SAM and NAD^+^ homeostasis through nicotinamide N-methyltransferase (NNMT), which methylates nicotinamide (a NAD^+^ precursor) using SAM, thereby simultaneously consuming methyl groups and reducing NAD^+^ availability (*42*). Since SIRT1-mediated circadian chromatin deacetylation depends on NAD^+^ (‘Epigenetic Regulation: Histone Modifications’), high NNMT activity could impair the clock through both reduced methylation potential and reduced SIRT1 activity. NNMT expression in the liver is itself circadian (*43–46*), but whether it constitutes a functional bridge between SAM metabolism and the NAD^+^/SIRT1 axis of the clock, remains to be established.

## Crosstalk Between One-Carbon Metabolism and the Circadian Clock

**Fig. 2:**
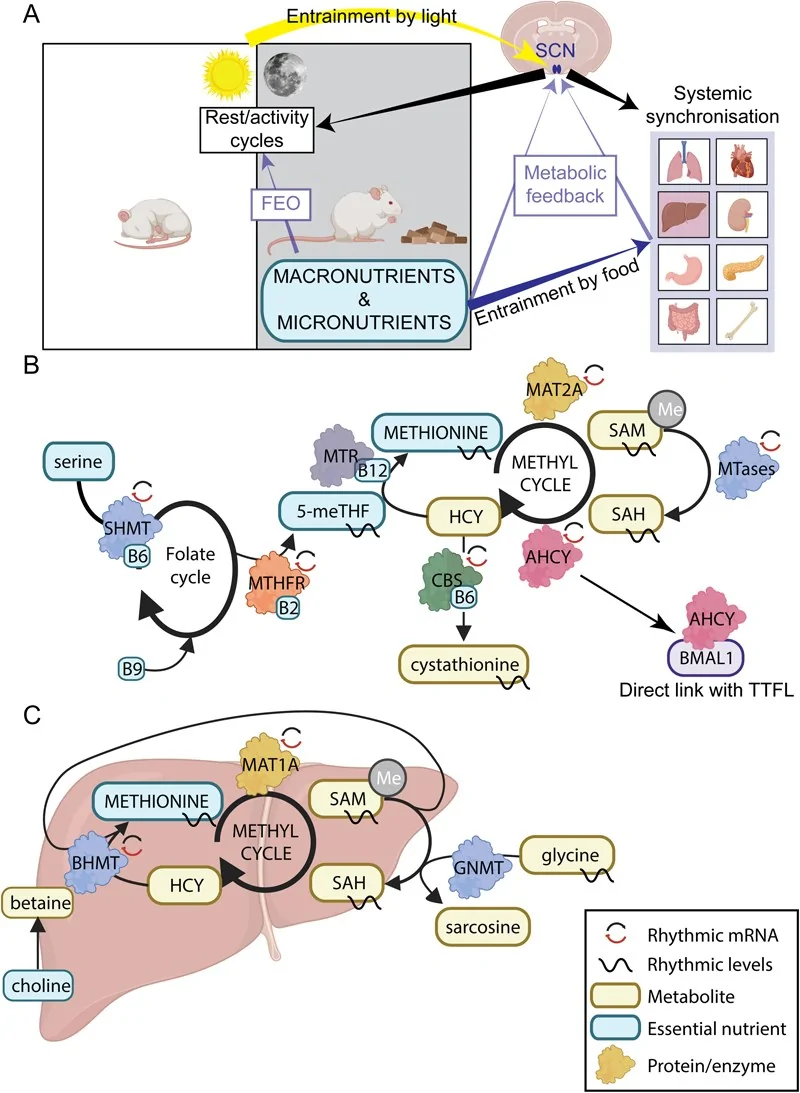
**Crosstalk between the circadian clock and one-carbon metabolism.** (A**)** Hierarchical organization and nutritional feedback. Circadian rhythms in mammals are orchestrated by a master pacemaker in the SCN of the hypothalamus, entrained primarily by the light–dark cycle via the retinohypothalamic tract. The SCN synchronizes peripheral clocks in organs including the liver and the gut through neuronal and humoral signals. Feeding, the principal non-photic zeitgeber, entrains peripheral clocks directly through the availability of macronutrients and micronutrients, independently of the SCN (*48**,*  *50*). The liver, as the principal site of SAM synthesis and methyl cycle activity, is particularly sensitive to nutritional entrainment. Food anticipatory activity, driven by a putative FEO that persists in SCN-lesioned animals, also contributes to the rest–activity cycle independently of SCN output (*48*). The entrainment hierarchy is not strictly top-down: peripheral metabolic state, directly from nutrients or from systemic factors, feeds back to the SCN. (B) The methyl and folate cycles and some liver-specific variations. (C) SAM is synthesized from methionine and ATP by MAT1A and MAT2A and donates its methyl group to over 200 acceptor substrates in reactions catalysed by methyltransferases (MTases), including DNA, histones, RNA and lipids, generating SAH as a by-product. SAH is hydrolysed to homocysteine (Hcy) by AHCY. Hcy is either remethylated to methionine via MTR (requiring vitamin B12) or BHMT (using choline-derived betaine in the liver), or enters the transsulfuration pathway (requiring vitamin B6) to produce cysteine and glutathione (GSH). The folate cycle provides the methyl group for remethylation, either directly from dietary 5-methyltetrahydrofolate (5-meTHF) or by reduction of vitamin B9 or natural folates that first acquire a one-carbon unit from serine via SHMT (requiring vitamin B6), and then are irreversibly reduced to 5-methylTHF by MTHFR (requiring vitamin B2). GNMT acts as a hepatic safety valve, consuming excess SAM by methylating glycine to sarcosine. Many key enzymes of the methyl cycle are rhythmically expressed in the liver and SAM itself oscillates in a circadian manner (*37**,*  *41**,*  *43–45*), coupling the nutritional state of the cell to the temporal regulation of methylation reactions. By directly associating with BMAL1, AHCY links the methyl cycle to the TTFL of the clock. Figure elements from BioRender. Fustin, J. (2026)

The relationship between nutrition, one-carbon metabolism and the circadian clock must be understood within the broader architecture of the mammalian circadian system, which is organized hierarchically but subject to regulation at multiple levels simultaneously (Fig. 2A). Light is the dominant zeitgeber for the SCN (*47*), which orchestrates peripheral clocks through neuronal projections and humoral signals including glucocorticoids and body temperature rhythms (*48*). However, feeding time acts as a largely SCN-independent zeitgeber for peripheral clocks: when food access is restricted to the rest phase, hepatic and other peripheral clocks shift by many hours while the SCN remains phase-locked to light (*48**,*  *49*). This dissociation reveals that the liver clock, the principal site of SAM synthesis and methyl cycle activity (*28*), is particularly responsive to nutritional entrainment signals (*50*). Beyond entraining peripheral clocks, feeding schedules also drive food anticipatory activity through a putative food-entrainable oscillator (FEO), a circadian timing mechanism that persists in SCN-lesioned animals and whose anatomical substrate, though not yet conclusively identified, likely involves distributed hypothalamic and limbic circuits (*47**,*  *48*). Crucially, this entrainment hierarchy is not strictly top-down: peripheral metabolic state feeds back to the SCN, as illustrated by the reprogramming of the SCN circadian transcriptome of mice fed a methyl-deficient diet (*51*) or high-fat diet (*52*).

One-carbon metabolism therefore potentially sits at a mechanistically significant intersection of this system: as both a target of feeding-driven entrainment in peripheral tissues and a potential source of metabolic feedback to the central clock, the methyl cycle is positioned to amplify or disrupt circadian organization at multiple levels of the hierarchy simultaneously.

### A bidirectional relationship

The crosstalk between the methyl cycle and the circadian clock operates in both directions and is explored here around three themes: the dependence of the clock on methyl group supply, the direct molecular interface between AHCY and BMAL1 at circadian gene promoters and the reciprocal clock-driven control of one-carbon metabolism. Fig. 2B and C integrate the metabolic pathways presented in ‘One-carbon Metabolism—the Biochemical Engine of Methyl Group Supply’ with the evidence presented in this section.

The strength of evidence for the crosstalk described in this review varies considerably across levels of biological organization. The direct physical interaction between AHCY and BMAL1 at chromatin, and the consequent loss of H3K4me3 upon AHCY inhibition, are supported by mechanistic data from biochemical, genetic and genome-wide approaches. By contrast, the relevance of this crosstalk to whole-animal physiology, behaviour and disease rests on correlative and associative evidence, and the translational extensions discussed in later sections are explicitly hypothesis-generating. We have aimed to signal this gradient of evidence throughout.

### Methylation is required for circadian timekeeping across evolution

The first evidence that methyl group availability is fundamentally required for circadian rhythm generation came from pharmacological experiments using 3-deazaneplanocin A (DZnep), an inhibitor of AHCY that prevents SAH hydrolysis, thereby causing SAH accumulation that competitively displaces SAM from methyltransferase active sites and globally suppresses transmethylation reactions (*53*). It should be noted that DZnep was originally characterized in the cancer biology literature as an EZH2 inhibitor, where it was shown to deplete EZH2 protein and reduce H3K27me3 in cancer cells through an indirect mechanism involving SAH-mediated inhibition of EZH2 chaperone methylation and consequent proteasomal degradation (*54*). The relationship between these two activities is therefore mechanistically intertwined, as the EZH2 effect is itself likely downstream of SAH accumulation rather than a direct off-target effect. The specificity of DZnep’s circadian action for the AHCY/SAH axis was nevertheless established by the 5′-methylthioadenosine/S-adenosylhomocysteine nucleosidase (MTAN) rescue experiment described below. Treatment of human and mouse cells expressing bioluminescent circadian reporters with DZnep produced a potent, dose-dependent lengthening of the circadian period (*53**,*  *55*). This effect was subsequently shown to be conserved across all eukaryotic clades tested (zebrafish, *Drosophila*, *Caenorhabditis elegans*, *Arabidopsis* and unicellular green algae) and a response, albeit weaker, was also detectable in cyanobacteria, which notably use a post-translational rather than transcriptional oscillator (*53*). The universality of this finding across organisms separated by more than 2.5 billion years of evolution strongly argues that the link between methyl metabolism and biological timing is a deeply ancient and fundamental feature of life (*37**,*  *53*).

The causal role of methyl cycle inhibition, rather than EZH2 depletion or any other DZnep target, was established by a molecular rescue experiment. Expression of the bacterial enzyme MTAN from *Escherichia coli* in mammalian cells provides an alternative route for SAH catabolism that bypasses AHCY entirely. Cells expressing MTAN were completely protected from DZnep-induced period lengthening even at saturating inhibitor concentrations, demonstrating that the clock phenotype depends specifically on SAH accumulation and the resulting methylation deficiency (*53*). Since MTAN has no known relationship to EZH2 activity or stability, its rescue of the circadian phenotype effectively rules out EZH2 depletion as the primary mechanism underlying the period-lengthening effect of DZnep in this context. Both histone methylation and m6A mRNA methylation were identified as the methyltransferase outputs most relevant to period control (*53**,*  *55*). Consistent with this interpretation, the Greco *et al.* study found that H3K27me3, the principal product of EZH2, was not significantly altered at circadian gene promoters upon AHCY inhibition (*33*), further supporting the view that the circadian consequences of methyl cycle perturbation are driven primarily through the SAM/SAH ratio and its effects on active histone and RNA methylation rather than through EZH2-dependent repressive chromatin. The contribution of different mRNA methyl marks to circadian timekeeping remains to be investigated *in vivo*.

An important complementary finding emerged from studies of exogenous SAM supplementation. Rather than shortening the period, excess exogenous SAM lengthened the circadian period in a dose-dependent manner. Metabolic tracing revealed the explanation: excess SAM is rapidly catabolized through the methionine salvage pathway to 5′-methylthioadenosine (MTA) and then adenine, both of which inhibit AHCY, causing paradoxical SAH accumulation and methylation deficiency (*56*). This counter-intuitive finding has direct clinical relevance given the widespread availability of SAM as a dietary supplement and illustrates that the circadian clock can serve as a sensitive physiological read-out of methyl cycle perturbation.

### AHCY as a molecular bridge: direct interaction with BMAL1 at chromatin

Beyond its enzymatic role in maintaining the SAM/SAH ratio, AHCY functions as a direct molecular component of the circadian transcriptional machinery. Mass spectrometry analysis of BMAL1 nuclear interactors in mouse liver revealed AHCY as a high-confidence BMAL1-associated protein, with the interaction peaking at ZT8—the phase of maximal CLOCK–BMAL1 transcriptional activity (*33*). Mapping of the interaction domain revealed that AHCY binds the PAS-B domain of BMAL1 (the same region involved in CLOCK dimerization) placing AHCY structurally at the heart of the transcriptional activation complex (*33*). Genome-wide ChIP-seq revealed that AHCY inhibition reduced the number of BMAL1 chromatin binding sites 4-fold specifically at the activating phase, while leaving CT12 binding largely unaffected (*33*). The conclusion is that AHCY, by locally hydrolysing the SAH generated as methyltransferases act at clock gene loci, sustains the permissive chromatin state required for productive BMAL1 engagement at E-boxes across the genome.

Among histone methylation marks examined at circadian gene promoters, H3K4me3 is selectively sensitive to perturbations of the SAM/SAH ratio. Upon AHCY inhibition or deletion, genome-wide H3K4me3 peaks at circadian gene promoters were strongly and specifically attenuated: 97% of the 389 changed peaks at CT24 were decreased and enriched specifically for circadian rhythm gene ontology terms (*33*). This selectivity at circadian loci is consistent with broader evidence that methionine restriction reduces global H3K4me3 levels while leaving H3K9me3 and H3K27me3 relatively unchanged (*39*). Loss of cyclic H3K4me3 upon AHCY inhibition was coupled with decreased oscillation of H3K9 and H3K14 acetylation, consistent with the hierarchical model in which MLL1-mediated H3K4me3 creates the permissive chromatin state required for subsequent CLOCK-mediated histone acetylation (*9**,*  *33*). Together, these findings define a regulatory circuit in which BMAL1 recruits AHCY to circadian gene promoters, where local SAH clearance enables MLL1 methyltransferase activity, sustaining the rhythmic H3K4me3 that is prerequisite for BMAL1’s own chromatin engagement in a feedforward loop coupling the clock activator and a methyl cycle enzyme at chromatin.

### The clock controls one-carbon metabolism

Systematic reanalysis of multitissue mouse circadian transcriptomics datasets showed that one-carbon metabolism was the second most significantly enriched gene ontology in the liver, immediately after circadian rhythm itself (*37**,*  *44*). Transcriptomics studies in the fasted liver further reveals that one-carbon metabolism enzymes are both regulated by the circadian clock and by nutrient availability and feeding/fasting cycles (*43**,*  *45*). While these transcriptional rhythms do not always translate into rhythmic total protein abundance, circadian phosphoproteomics datasets provide evidence that many of these enzymes are indeed regulated in a circadian manner (*46*). At the metabolite level, hepatic SAM peaks during the subjective day (around CT4) while SAH and related metabolites oscillate with distinct phase relationships, suggesting that the methylation potential itself is highest in the early active phase in mice (*37**,*  *41*). Circadian rhythms in histone methylation, DNA methylation and mRNA m6A levels have also been observed, though their mechanistic connections to the oscillating SAM pool remain to be fully elucidated (*15**,*  *33**,*  *57*).

Importantly, this clock-driven regulation of methyl metabolism is not unidirectional, and nutritional state actively reshapes the same transcriptional programmes. Recent high-resolution atlases of the fasting mouse liver reveals that, beyond their circadian rhythmicity, *Mthfr*, *Mat1a* and *Ahcy* are additionally regulated at the level of expression and/or alternative splicing in response to fasting (*43**,*  *45*). That these core methyl cycle enzymes are jointly gated by the molecular clock and by nutritional state, and at two independent layers of gene regulation, further highlights the degree to which methyl group supply in the liver is an integrative read-out of both temporal and dietary signals.

### Dietary disruption of the methyl cycle disrupts the clock in vivo

The strongest *in vivo* demonstration of the methyl cycle–clock connection in a nutritional context comes from studies using the methionine–choline deficient (MCD) diet. Mice fed an MCD diet showed profound disruption of circadian locomotor activity rhythms, with a shorter circadian period, *i.e.* a faster clock, in constant conditions (*51*). Moreover, *Mat2a*, the ubiquitous isoform of MAT, was the single gene most significantly gaining rhythmicity in the SCN of MCD-fed animals, suggesting a specific nutrigenomic signal relaying systemic deficiency to the master clock (*51*). 1C metabolites in both liver and SCN were altered in a circadian time-dependent manner, further demonstrating the crosstalk between one-carbon metabolism and the circadian clock.

It is important to note, however, that the MCD diet is a nutritional stressor causing substantial weight loss, hepatic injury, inflammation and systemic sickness behaviour, each independently capable of affecting locomotor activity. The behavioural and transcriptomic changes observed under MCD feeding therefore cannot be attributed specifically and exclusively to methyl donor deficiency; they may equally reflect these broader physiological consequences of the diet. However, several observations argue against a purely non-specific interpretation. First, the most prominent effect of the MCD diet was a shortened free-running circadian period in constant darkness, *i.e.* a direct behavioural read-out of the endogenous SCN clock, rather than a parameter such as overall activity level or rhythm amplitude that would be more readily attributable to general ill health. Second, this effect disappeared within days of returning to a normal diet, while weight loss and hepatic steatosis persisted, indicating that these metabolic sequelae were not themselves the cause of the circadian phenotype. Third, the specific gain of circadian rhythmicity by Mat2a in the SCN represents a directionally specific molecular change not readily predicted by generic sickness behaviour or weight loss. Nevertheless, disentangling the contribution of dietary methyl donor deficiency per se from these broader physiological confounds will require models that more selectively restrict methionine or SAM availability without inducing comparable systemic injury, weight loss or inflammation.

### Knowledge gaps and open questions

Several fundamental questions remain unanswered. First, whether the clock controls AHCY recruitment through PTMs of BMAL1, or whether their interaction is constitutive with periodicity driven purely by the oscillating chromatin landscape and/or AHCY expression, is unresolved. Second, whether circadian oscillations in hepatic SAM reflect direct CLOCK–BMAL1 transcriptional control of methyl cycle enzymes or are an indirect consequence of feeding/fasting rhythms has not been cleanly disentangled. Third, how day-to-day variation in dietary methyl donor intake translates into changes in clock amplitude, period or phase has not been measured: serum methionine concentrations in healthy humans vary across a range sufficient to alter histone methylation *in vitro* (*39*), suggesting common dietary variation could have cumulative chronobiological effects. Finally, the relative contributions of RNA methylation (m6A, cap methylation) versus histone methylation to clock phenotypes induced by methyl cycle perturbation remain incompletely resolved. Addressing these gaps will require tissue-specific conditional genetic models, metabolic isotope tracing across the circadian cycle and carefully controlled dietary intervention studies.

## Implications for Health and Disease

The sections below describe the coexistence of one-carbon metabolic dysregulation and circadian disruption across several disease contexts. In most cases, the directionality of this relationship, whether metabolic dysfunction drives circadian disruption, the reverse, or both arise as parallel consequences of a shared upstream insult, has not been experimentally established, and we make this explicit throughout. Similarly, the therapeutic implications discussed are hypothesis-generating rather than evidence-based recommendations.

### Liver disease: where methyl deficiency and circadian disruption converge

The liver is both the primary site of SAM metabolism and a major peripheral clock organ, making it uniquely susceptible to pathology arising from perturbation of either system. The MCD diet model—which depletes both principal dietary methyl donors and reproducibly causes steatohepatitis, inflammation and fibrosis—illustrates how methylation deficiency disrupts not only hepatic lipid metabolism but the entire circadian transcriptome of the organ (*51*). The loss of phosphatidylcholine synthesis via the phosphatidylethanolamine N-methyltransferase (PEMT) pathway under methionine deficiency impairs mitochondrial membrane integrity, depletes mitochondrial glutathione and activates ER stress—a constellation of injuries that is markedly worsened by circadian misalignment (*58*). Conversely, loss of the liver-specific MAT isoform MAT1A, either genetically in mice or through epigenetic silencing in human non-alcoholic fatty liver disease, causes chronic SAM deficiency, which drives global DNA hypomethylation, oxidative stress and spontaneous progression to HCC (*28*). The pathological MAT1A → MAT2A isoform switch that occurs in liver injury and cirrhosis further reduces SAM availability, establishing a vicious cycle in which impaired methylation capacity promotes the dedifferentiated, proliferative state of transformed hepatocytes (*28*). While the MAT1A → MAT2A switch is primarily driven by disease-associated loss of hepatocyte identity rather than by circadian disruption per se, the nutritional context in which it occurs, *i.e.* methyl deficiency, is itself sufficient to perturb circadian organization, as evidenced by the *de novo* circadian oscillation of *Mat2a* in the SCN of methyl-deficient mice (*51*). The MAT1A → MAT2A switch should therefore be understood as a metabolic adaptation to liver disease that shares an upstream trigger (dietary methyl insufficiency) with circadian disruption, rather than as a direct consequence of clock dysfunction.

The circadian dimension of this hepatic pathology is increasingly appreciated. NASH and liver fibrosis are exacerbated by circadian misalignment in rodent models, and clock gene disruption accelerates steatohepatitis progression (*51**,*  *59*). Given that the MCD diet profoundly reprograms the hepatic circadian transcriptome, with thousands of genes gaining or losing rhythmicity, and core metabolic regulators such as Ppara, Nampt and Mat2a showing altered circadian profiles (*51**,*  *59*), it is plausible that circadian disruption contributes to the metabolic and inflammatory pathology of methyl-deficient liver disease, although direct causal evidence for this contribution, as opposed to co-occurrence driven by a shared upstream cause, is currently lacking.

### Cancer: methylation, clock silencing and SAM

Aberrant DNA methylation is a hallmark of cancer, and the connections between one-carbon metabolism, SAM availability and cancer epigenetics are extensive. Diets deficient in methyl donors promote hepatocarcinogenesis in rodents through a stereotyped sequence of epigenetic events: global genomic hypomethylation (which activates proto-oncogenes), gene-specific hypermethylation (which silences tumour suppressors) and altered histone methylation at regulatory loci (*38*). Conversely, SAM has demonstrated antiproliferative, proapoptotic and antimetastatic activity in liver cancer cell lines, selectively reversing the hypomethylation of metastasis-promoting genes while leaving primary hepatocytes largely unaffected (*60*).

The circadian clock is itself a tumour suppressor system: rhythmic expression of PERs and BMAL1 restrains cell proliferation and DNA damage responses, and disruption of this rhythmicity promotes oncogenesis (*61*). Promoter hypermethylation of PERs, CRY1 and BMAL1 has been documented in breast cancer, chronic myeloid leukaemia, endometrial cancer and haematological malignancies and correlates with poor prognosis (*16*). This creates a potential feedforward relationship in which methyl cycle deficiency, whether from dietary insufficiency, genetic polymorphism or pathological MAT1A silencing, simultaneously impairs both the direct tumour-suppressive activity of SAM-dependent methylation and the indirect tumour suppression afforded by a functional circadian clock, although whether these two impairments interact synergistically *in vivo*, or simply co-occur as parallel consequences of the same metabolic insult, has not been tested. Whether restoring SAM levels or correcting circadian alignment can break this cycle in established tumours remains a clinically important open question.

It should be noted that the relationship between SAM and cancer is not simply that more SAM is better. As discussed in ‘Methylation is Required for Circadian Timekeeping across Evolution’, exogenous SAM at supraphysiological concentrations paradoxically inhibits methylation through its catabolism to MTA and adenine, which inhibit AHCY (*56*). Furthermore, in liver cancer cells, SAM treatment produces both hypo- and hypermethylation depending on the locus (*60*), indicating that the epigenomic consequences of SAM supplementation are context- and dose-dependent, and that the SAM/SAH ratio rather than absolute SAM levels is the relevant physiological determinant.

### Aging, polyamines and the deteriorating clock

Both the circadian clock and the methyl cycle show functional decline with aging, and there is emerging evidence that these trajectories are mechanistically connected. Aged mice show lengthened and less robust circadian locomotor activity rhythms (*62*), and this phenotype is partially reproduced by AHCY inhibition in the SCN (*33**,*  *55*), consistent with the hypothesis that declining AHCY activity during aging may contribute to circadian deregulation through SAH accumulation (*33*). In *Drosophila*, Ahcy depletion reduces lifespan and causes an aging phenotype characterized by SAH accumulation (*63*), establishing a genetic link between methyl cycle enzyme activity and longevity.

The polyamine–clock connection provides another mechanistic thread. Spermidine and spermine, synthesized from decarboxylated SAM, display circadian oscillations in mouse liver and have been shown to regulate circadian period through a mechanism that declines with age (*64*). Since polyamine synthesis represents a major competing sink for SAM, its circadian regulation means that the demand on SAM, and thus its availability for histone and DNA methylation, fluctuates across the day in an age-sensitive manner. Methionine restriction, which extends lifespan in multiple model organisms, does so at least in part through its effects on SAM-dependent methylation and autophagy regulation (*65*), and the circadian dimension of this response, including the possibility that methionine restriction acts partly by resetting or amplifying circadian oscillations, has not yet been systematically investigated.

### Neurological and psychiatric disorders

One-carbon metabolism and circadian rhythms converge on neurological disease through multiple pathways. Hyperhomocysteinaemia, arising from folate, B12, or B6 deficiency or MTHFR polymorphisms, is associated with cognitive impairment, depression and increased risk of Alzheimer’s disease (*66*). An MTHFR polymorphism has been linked to chronic insomnia (*67*), providing a direct genetic connection between 1C enzyme function and sleep/circadian disturbance. SAM has been investigated as an antidepressant. The finding that exogenous SAM affects circadian locomotor activity in mice (*56*) suggests that the chronobiological effects of SAM supplementation may confound or contribute to its reported effects on mood, an interaction that clinical trials have not yet adequately controlled for. More broadly, the SCN itself is sensitive to methyl cycle perturbation: the MCD diet induces *de novo* circadian oscillation of *Mat2a* specifically in the SCN, suggesting that the master clock actively responds to systemic methyl metabolite status as a nutritional entrainment cue (*51*).

### Therapeutic and nutritional implications

The evidence reviewed here carries several practical implications. First, the time-of-day at which methyl donor status is assessed matters: SAM, SAH, methionine and related metabolites oscillate across the circadian cycle in both liver and brain, meaning that samples collected at different times of day will yield systematically different results (*37**,*  *51*). This confound likely contributes to apparent inconsistencies in the literature on MCD diet effects on hepatic methylation status. Second, dietary interventions targeting one-carbon metabolism, including folate supplementation, methionine restriction, choline supplementation and SAM administration, are likely to have chronobiological consequences that have not been considered in their design. Third, the circadian clock may represent a sensitive and quantitative read-out of methyl cycle sufficiency, as period length responds proportionally to the degree of methylation deficiency (*53*). While highly speculative at present, this raises the possibility that wearable circadian monitoring, increasingly considered as a way to measure the circadian time of patients in personalized medicine (*68**,*  *69*), could also eventually serve as a non-invasive proxy for methyl metabolic status in clinical populations, a hypothesis that would require validation against direct metabolite measurements before any clinical application could be considered. Fourth, given that SAM exerts its effects through the SAM/SAH ratio rather than through absolute SAM abundance, interventions that reduce SAH (e.g. by supporting AHCY activity through adequate adenosine clearance) may be as effective as those that increase SAM supply.

## Conclusion

The evidence presented in this review establishes that one-carbon metabolism and the mammalian circadian clock are not merely coregulated physiological processes but are mechanistically intertwined at the molecular, cellular and systemic levels. SAM, the universal methyl donor produced by the folate and methionine cycles, is an essential substrate for the histone and RNA methylation events that constitute the circadian epigenome and epitranscriptome. The AHCY–BMAL1 interaction at circadian gene promoters represents a direct and specific molecular interface through which methyl group supply is coupled to clock-driven transcription, revealing that the clock activator itself recruits a methyl cycle enzyme to sustain the chromatin state required for its own function. In the opposite direction, the clock transcriptionally orchestrates the expression of 1C metabolism genes and creates circadian oscillations in SAM and SAH that temporally gate the methylation potential of the cell. Dietary perturbation of this system, through deficiency of methionine, choline, folate or B vitamins, or through supraphysiological SAM intake, profoundly disrupts circadian behaviour and reprograms the circadian transcriptome in both peripheral tissues and the SCN.

These findings have implications for our understanding of metabolic liver disease, cancer, aging and neurological disorders, all of which involve both methylation dysregulation and circadian disruption. A critical priority for future research is to determine the causal chain from dietary methyl supply to circadian function to pathological outcome, work that will require tissue-specific genetic models, metabolic isotope tracing at multiple circadian time points and human intervention studies designed with chronobiological rigour. The circadian clock, long studied as a transcriptional oscillator, is increasingly revealed as a metabolic sensor with ancient roots in prebiotic chemistry that connects the nutritional environment of the organism to the temporal organization of its genome.
